# The use of dietary isotopes as a preliminary step in the death investigation of unidentified skeletal human remains in British Columbia, Canada

**DOI:** 10.1111/1556-4029.15653

**Published:** 2024-11-07

**Authors:** Damon Tarrant, Laura Yazedjian, Joe Hepburn, Stephen Fonseca, Sahra Talamo, Michael Richards

**Affiliations:** ^1^ Department of Archaeology Simon Fraser University Burnaby British Columbia Canada; ^2^ British Columbia Coroners Service Burnaby British Columbia Canada; ^3^ International Committee of the Red Cross African Centre for Medicolegal Systems Pretoria South Africa; ^4^ Department of Chemistry G. Ciamician, Alma Mater Studiorum University of Bologna Via Selmi 2 Bologna Italy; ^5^ Department of Human Evolution Max Planck Institute for Evolutionary Anthropology Leipzig Germany

**Keywords:** British Columbia, Canada, death investigation, dietary isotopes, forensic anthropology, medicolegal significance, stable isotopes

## Abstract

In British Columbia, Canada, unidentified skeletal human remains are often recovered by law enforcement or civilians and there is a question if they are modern and of medicolegal significance, or historical or archaeological. We used relatively fast and inexpensive carbon and nitrogen stable isotope analysis of human bone collagen from a selection of these remains (*n* = 48) combined with a logistic regression model to classify remains as modern, historical, or archaeological. We then confirmed our temporal classification through directly radiocarbon dating each individual and found that we could predict the temporal group with 93% accuracy. In regions where archaeological remains have dietary isotope values distinct from living people, dietary stable isotope analysis can provide a time‐, and resource‐efficient method to screen cases of unidentified human remains early in death investigation.

**Highlights**

- We use isotope analysis to determine if unidentified human remains are modern or not.
- Isotope methods were accurate in determining if the remains were modern or historic/archaeological.
- We confirmed our findings with radiocarbon dating.

## 1 INTRODUCTION

Archaeological skeletal remains are often well preserved in British Columbia (BC) due to unique burial conditions (i.e. shell middens); therefore, standard anthropological techniques cannot always inform whether these remains are archaeological or recent in age, and therefore of potential forensic interest. Currently, there are approximately 180 open cases with the BC Coroners Service involving unidentified human remains in British Columbia. Determining which remains are archaeological or modern is important in BC as archaeological remains need to be returned to their ancestral communities, and forensic cases need to have greater resources and time invested into them by the Coroners Service for identification.

This paper seeks to demonstrate how carbon and nitrogen isotopes of human bone collagen can be used in death investigation, especially in the context of British Columbia. Stable isotope analysis has been used to reconstruct the diet and migration of individuals in archaeology, ecology, and forensic science [1, 2, 3, 4, 5, 6]. Carbon, nitrogen, and sulfur isotopes are the most widely applied isotope systems for dietary studies [1, 4, 7, 8, 9, 10, 11]. Here, we present the results of our application of isotope analysis to 48 unidentified human remains under the jurisdiction of the BC Coroners Service to help establish whether in this jurisdiction isotope analysis can be used as a relatively rapid and inexpensive screening method to identify whether these remains are archaeological or more recent (historic or of forensic interest). This project has been undertaken in collaboration with the British Columbia Coroners Service which oversees death investigation within the province.

### 1.1 Dietary isotope analysis of carbon and nitrogen

**FIGURE 1:**
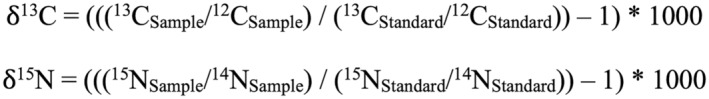
Delta notation equations for carbon and nitrogen isotope ratio values.

A complete review of isotopic methods is outside the scope of this paper; for broader reviews, please see the references cited here [4, 6, 10, 12, 13]. Briefly, carbon and nitrogen isotopic studies of human tissues are the most fundamental isotope systems for dietary reconstruction. Through both carbon and nitrogen, archaeologists and anthropologists are able to determine whether an individual had a diet relying on terrestrial protein (such as cattle or chicken), fresh water protein, or marine protein, and whether the local food web was based in a C_3_, or C_4_ plant system [1, 7, 8, 14, 15]. When dealing within unidentified human remains, a fundamental understanding of contemporary diet, and diet in the archaeological past can be useful to determine whether remains are of forensic interest, and which are not. Carbon and nitrogen isotope values are presented in delta notation using the formula given in Figure 1.

Carbon isotopes enter the food web in plants during the aspiration of CO_2_ [1, 4]. There are two major photosynthetic pathways for plants that are relevant for forensic investigations, C_3_ and C_4_ [4, 6, 15]. The vast majority of native plants within British Columbia are C_3_ plants [1, 16]. However, for warmer and more arid regions, there is a greater chance of C_4_ plant dietary reliance. C_3_ plants have *𝛿* ^13^C values of −35‰ to −20‰ as they discriminate against the heavier carbon‐13, and C_4_ plants have *𝛿* ^13^C values of −14‰ to −10‰ as they incorporate more of the heavier carbon‐13 relative to the lighter carbon‐12 [15]. Thus, carbon isotopes can be used to differentiate whether a population had a food web based on C_3_ or C_4_ plants, or a mix of both. Considering human diet, C_3_ plants are most fruits and vegetables, rice and wheat, while C_4_ plants that are consumed by people include sugarcane, millet, and maize [6, 15]. Between diet and human consumers’ bone collagen, there is a fractionation of approximately *𝛿* ^13^C ~5‰, meaning that the consumers’ collagen is on average ~5‰ higher than their diet [17]. Importantly, the carbon dissolved in the ocean contains much more of the heavier carbon‐13, resulting in higher *𝛿* ^13^C values for plants and animals within the oceans [1, 18]. Therefore, when considering carbon isotopes on their own, there may be some ambiguity as to whether an individual was consuming marine proteins, or terrestrial proteins based on C_4_ plants.

Like carbon isotopes, nitrogen is first introduced to the food web through plants as most take up bioavailable nitrogen from the soil [19]. The ratios of plant nitrogen isotopes are also a function of the local environment; in dryer and hotter regions, the baseline nitrogen ratios of plants are enriched [19, 20]. Furthermore, fertilization and manuring of crops can result in higher *𝛿* ^15^N values [19, 21]. Therefore, to contextualize nitrogen ratios, a baseline for the specific environment should be constructed. Between diet and consumer, nitrogen fractionates on average *𝛿* ^15^N ~3‰ [22]. Through this fractionation, there is a trophic level effect, whereby it is possible to infer the trophic position of an individual (if a nitrogen baseline has been effectively constructed) [22]. This is useful when examining populations that may be consuming high levels of marine protein as oceanic food webs have more trophic levels than terrestrial food webs. Nitrogen and carbon isotopes can be used to determine whether an individual had a terrestrial C_4_ diet or a diet with high levels of marine protein [22].

### 1.2 First Nations’ diets prior to colonization in British Columbia

The Indigenous peoples of coastal British Columbia often maintained a diet based on high levels of marine protein such as seal, sea lion, or salmon [1, 23]. Without question, they also consumed plant foods readily; however, as bone collagen is constituted from the dietary protein, plant food information is often masked by the dietary protein signature [24]. Schwarcz et al. (2014) found the average *𝛿* ^13^C value of coastal populations to be −13.1‰ ± 0.6‰, and the average *𝛿* ^15^N to be 18.6‰ ± 1.2‰. This supports that individuals living in coastal regions have diets consisting of high trophic‐level marine protein. However, interior populations likely maintained an intermediary isotopic values between marine and terrestrial as salmon migration would maintain an oceanic signal, and a greater influence from C_3_ terrestrial resources.

### 1.3 Diets in modern British Columbia

Within Canada, there are no contemporary dietary studies of human bone collagen. However, studies using human hair suggest that the average diet for a contemporary Canadian individual is terrestrial [25]. Bone collagen is a representation of diet over many years of life and varies depending on which bone is sampled [4], whereas each centimeter of a hair sample generally reflects the diet equivalent to the month prior to growth [26, 27]. Human hair and bone collagen isotope values are therefore not directly temporally analogous. However, they do provide a rough expected range for contemporary human tissues. Bataille et al. [25] found that in human hair samples from across Canada the expected range for *𝛿* ^13^C values were −20.3‰ to −16.7‰, and *𝛿* ^15^N values wer 7.6‰ to 10.8‰. Using the offset from [28], we should expect modern bone collagen to be comparable to *𝛿* ^13^C = −18.9‰ to −15.3‰, and *𝛿* ^15^N = 8.5‰ to 11.7‰.

### 1.4 Theoretical background and hypothesis

This project takes a similar approach to Bartelink et al. [29], and Bartelink and Chesson [6], where dietary isotopes are used to separate between two populations, and determine which cases are of forensic interest, or not. As archaeological populations in British Columbia often had diets based on marine proteins, and contemporary populations maintain terrestrial diets, we hypothesize that stable isotope analysis can be effectively used to estimate whether unidentified human remains are archaeological or of forensic interest. Importantly, our hypothesis is not to provide conclusive evidence that a case is of forensic interest using only carbon and nitrogen isotope values, but rather, that carbon and nitrogen isotopes can be used to screen which cases are archaeological in context; allowing these remains to be expediently returned to community, and that government resources can be better directed to cases that are of medicolegal significance.

## 2 METHODS

### 2.1 Collagen preparation

Samples were prepared at three different isotope laboratories but all three use the same modified Longin method [30] outlined in Richards and Hedges [9] with the addition of an ultrafiltration step [31, 32]. In summary, human bulk cortical bone samples were first cleaned on their outer cortical surfaces using a Dremel drill, and then approximately 200 mg of either whole bone or bone powder was removed for isotopic analysis. This cleaning was done to remove any contamination from the burial environment (e.g., adhering soils), and any visibly contaminated areas prior to acid digestion [33]. Then samples were soaked in a weak (0.5 M) hydrochloric acid at 5°C until the bone was demineralized (as assessed through visual inspection). For exceptionally well‐preserved samples (i.e., ones that subsequently were found to be modern), demineralization at 5°C was not adequate, so these samples were demineralized at room temperature. After demineralization, the solute was gelatinized in acidic water (HCl pH 3) at 75°C in a heat block, to denature the collagenous protein. Samples were then filtered using a 5‐um Ezee Filter™ to remove remaining contaminates. Then, the samples were centrifuged in 30 kDa filters to isolate the >30 kDa fraction (following Brown et al. [31]). Prior to use, the Eeze filters were precleaned in de‐ionized water and the ultrafilters were cleaned through repeated steps of washing and ultrasonication with ultrapure water to eliminate any potential glycerol contaminant [32]. Samples were then frozen and lyophilized. Then, samples were weighed into tin capsules for subsequent isotope measurements. Following the findings of Liden et al., [34] we did not use NaOH in the sample preparation steps as we used ultrafiltration.

### 2.2 Standard reference materials and delta notation

Samples in this study were analyzed at three different labs over the last 14 years. These were at the former Department of Human Evolution, Max Planck Institute for Evolutionary Anthropology (Leipzig, Germany), the University of British Columbia Department of Anthropology (Vancouver, Canada), and Simon Fraser University Department of Archaeology (Burnaby. Canada). All isotope data were standardized to international standards VPDB for 𝛿^13^C, and AIR for 𝛿^15^N. Standard reference materials and equipment differed among the three labs and the equipment and standards used to determine the collagen isotope data are outlined in detail in Data S1. All samples were measured in duplicate. Sample information is given in Data S2.

As the samples were prepared in three different laboratories over a 14‐year time period, it is worth considering the comparability of data among the three labs. All three labs measured samples compared to international isotope standards and ran in‐house laboratory standards in each sample run. The errors on the internal standards for all three labs were better than 0.2 ‰ so we are confident in the data from each lab. Pestle et al. [35] carried out an interlaboratory comparison between a number of laboratories (including the Max Planck laboratory used here). In that study, there was a large difference between laboratories that used different preparation methods specifically for oxygen isotopes. However, in that study, the difference between labs for collagen carbon and nitrogen isotope values was much smaller, with the range for δ^13^C being 1.8‰ and for δ^15^N it was 1.9‰. These variations include some laboratories that were clear outliers (not the Max Planck laboratory). In this paper, they advocate for the use of minimum meaningful difference (MMD) measurements to determine the difference between labs. Unfortunately for our study, two of the laboratories no longer exist (MPI and UBC), so we could not carry out a test measuring the same sample in all three labs. However, given that the samples were prepared in the same way in each laboratory and repeat long‐term measurements of the internal standards in each lab were less than 0.2 per mil, we are confident that there is very little variation in values among the three labs. However, it is worth mentioning that even with the very extreme differences (1.8‰ for C and 1.9‰ for N) that were seen in the Pestle et al. [35] paper between labs using different preparation methods, it would not impact the main findings of our paper. As discussed below, the differences between the groups we identify are much larger than even the variation observed in Pestle et al. [35].

### 2.3 Quality indicators

For extracted bone collagen, the sample quality is generally inferred using the carbon to nitrogen ratio, and the % weight carbon and nitrogen [36, 37]. All samples had C:N ratios between 3.0 and 3.4 which is well within the generally accepted range of 2.9 and 3.6 for acceptable collagen [36]. The %wt carbon ranged from 30.7% to 53.9%, and the %wt nitrogen ranged from 11.2% to 17.2%, again within accepted ranges for well‐preserved collagen.

### 2.4 Radiocarbon dating

Over a period of 14 years, samples within this study were dated at three different labs. We dated 24 samples at the MAMS at Mannheim Germany, 2 at the University of Arizona AMS lab, and 23 at the University of Ottawa radiocarbon lab. The samples dated at Mannheim were first prepared for the collagen extraction in the Department of Human Evolution at the MPI, Leipzig, Germany, following the procedure described in the studies cited herein [32, 33]. In the two other cases, bone was sent directly to the laboratories for preparation and analysis. Radiocarbon dates for each sample are given in Data S2.

As our goal was to determine whether remains are of forensic interest, we did not calibrate the radiocarbon results or correct for the marine reservoir effect. This is because the modern samples have the significant input of radiocarbon from nuclear bomb testing, whereas the archaeological and historical samples did not (as indicated by the “F” notation for the dates given in Data S2). Calibrating the archaeological and modern samples would not have possibly led to their inclusion in the “modern” category, as that category is only for samples that have excess bomb radiocarbon as clearly identified through radiocarbon dating. As our goal is to screen cases early on in death investigation to ensure that they are not archaeological, the exact year of death is not necessary to validate our hypothesis. Instead, categorical determinations of “archaeological” or “contemporary” are all that are required.

### 2.5 Statistical analysis

Statistical analysis was done using R version 2023.12.0 + 369. Within the logistic regression, the sample was divided into a training sample = 44, and a test sample = 4. The samples were chosen randomly.

#### 2.5.1 Independent variables

The dataset in Supplementary Information Data has been scrubbed of any identifying information of the individual cases—including the case number, bone element, and the Police department associated with the case. However, we have coded whether the cases are from the interior or coastal cities in British Columbia. Cases reported by Police departments from interior regions have been coded as 1, and cases from coastal regions have been coded as 0. The remaining variables are the *𝛿* ^13^C, and *𝛿* ^15^N values for each case.

Importantly, the respective police departments cannot be assumed to be directly reflective of where an individual lived prior to death, as these investigations often include remains that have been removed from their original context. The interior or coastal variable attempts to provide some geolocational context, as interior or coastal; however, ultimately, it may not be accurate.

#### 2.5.2 Dependent variable

The radiocarbon dates, error, and laboratory ID for each sample are presented in supplementary information. Radiocarbon dates are not directly integrated into the statistical analysis, as modern samples do not have raw radiocarbon dates. Furthermore, radiocarbon dating is used to evaluate the validity of our hypothesis. To properly assess our research question, we have created two variables from the radiocarbon dates. First, we created a categorical variable termed “conclusion” that classifies each case as archaeological (prior to ~1850 AD), historic (1850–1950 AD), and forensic (modern). This “conclusion” variable is used for graphical presentation of data. A second binomial variable was used as the dependent variable in the logistic regression. It combines forensic, and historic groups and codes these samples as 0, and archaeological samples as 1. This binomial variable “conclusion binary” allows us to create a probabilistic model to estimate quantitatively which samples are not of forensic interest.

As our hypothesis is to estimate which remains are of forensic interest or need further testing (such as absolute dating), combining historic and forensic decreases the probability of a false‐negative result (a forensic case being identified as archaeological). However, it does increase the probability of a false‐positive result (an archaeological case being identified as forensic or historic). Importantly, this same limitation applies to the visual analysis of data without a probability estimate.

#### 2.5.3 Logistic regression

This paper seeks to present both how isotope analysis can be informative, with and without advanced statistical analysis. First, we present the scatterplot of *𝛿* ^13^C and *𝛿* ^15^N values for the samples, then we follow this with a logistic regression model. The intent of this model is to provide a probability method of estimating whether a sample is of forensic interest. Importantly, Shapiro–wilk tests of normality, QQ‐plots, and density plots were generated to determine whether the carbon and nitrogen values are a normal or bimodal distribution. Prior to radiocarbon dating, we anticipated that our sample would contain at least 1–2 populations, and thus the likelihood of bimodal distributions within the data increases. Logistic regression was selected as it makes no assumptions about the distributions of the data. While visual analysis of data will often provide enough information to determine which samples are not of forensic interest, this model provides a more objective method of coming to a similar conclusion.

## 3 RESULTS

**FIGURE 2:**
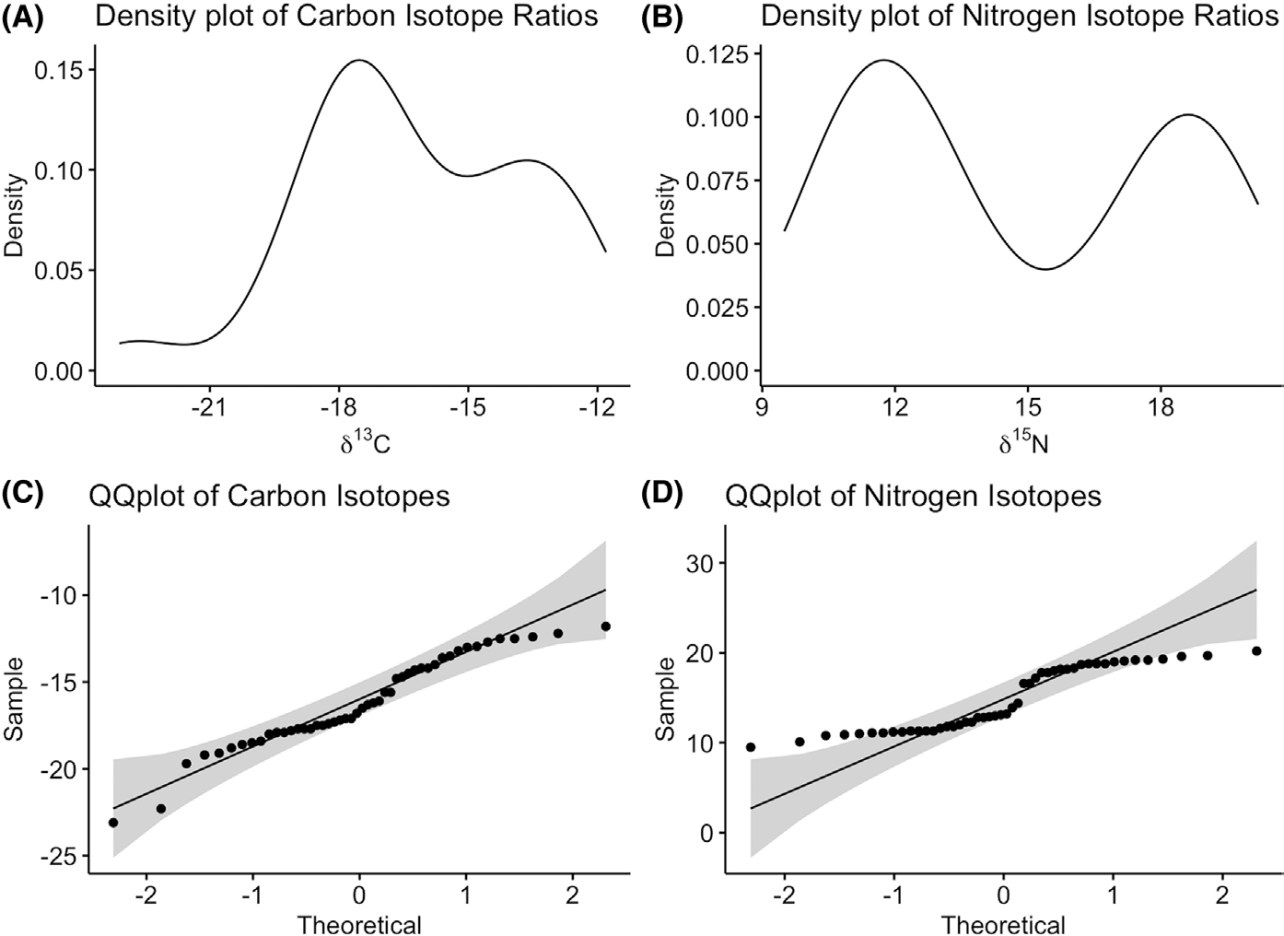
Quantile–quantile plots, and density plots of carbon (A & C) and nitrogen (B & D) isotopes values.

The entire sample's descriptive statistics can be found in Table 1. However, there are two theoretical populations within the sample. There was a large distribution in the *𝛿* ^13^C and *𝛿* ^15^N values supporting that there was a variety of subsistence patterns within the sample. The Shapiro–Wilk test of carbon isotopes values determined that it is normally distributed (w = 0.95495, *p* = 0.06312). This is further supported by the density and the QQplot in Figure 2. Nitrogen isotope values do not follow a normal distribution (w = 0.85613, *p* = 3.175e^−05^). Using the Mclust package [38], the mean of the modes was calculated, major mode = 11.85‰, and the minor mode = 18.53‰.

**TABLE 1:** Descriptive statistics of carbon and nitrogen isotope values of human bone collagen.

	Min	1st Q	Median	Mean	3rd Q	Max	SD
*𝛿* ^13^C ‰	−23.1	−17.8	−16.7	−16.2	−14.2	−11.8	2.62
*𝛿* ^15^N ‰	9.5	11.3	13.2	14.8	18.4	20.2	3.51
*𝛿* ^15^N ‰ major mode				11.85			
*𝛿* ^15^N ‰ minor mode				18.53			

**FIGURE 3:**
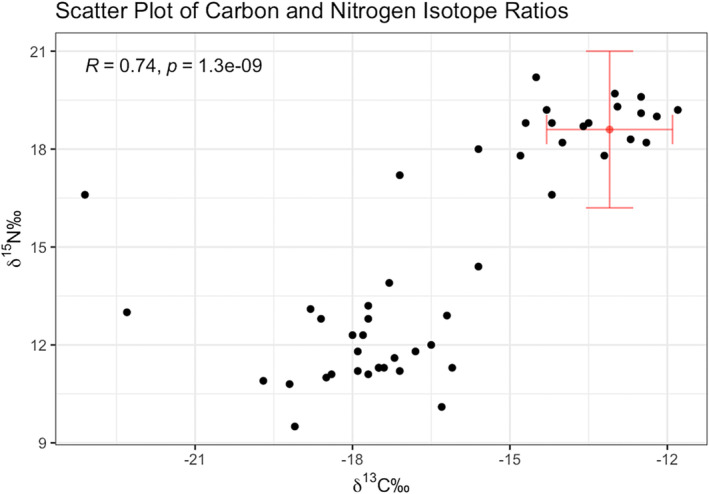
Scatterplot of carbon and nitrogen isotope values of human bone collagen. Samples from this study are in black, and the red cross indicates the expected values for archaeological populations (two standard deviations) from Schwarcz et al. 2014.

Figure 3 is a scatterplot of the sample data (in black), and the average carbon and nitrogen values for coastal archaeological populations in red (with two standard deviations) from Schwarcz et al. (2014). From this scatter plot, we see two clusters forming, one that is similar to Schwarcz et al.'s (2014) results (a marine diet), and a secondary cluster with lower nitrogen and carbon isotopes values, more similar to those from Bataille et al.'s hair isotope data (suggesting a terrestrial diet). Importantly, archaeological populations in the interior of British Columbia likely had a terrestrial or freshwater diet as marine resources would not be as accessible. There are two samples (Study IDs 1 and 25) had *𝛿* ^13^C values below −21‰, and *𝛿* ^15^N values above 12‰. We suggest that these two samples will be archaeological in nature and had a diet with heavy contributions from freshwater fish. In line with our hypothesis, we argue that the samples clustering around *𝛿* ^13^C = −18‰, and *𝛿* ^15^N = 12‰ will be of a more recent context and may be of forensic interest.

### 3.1 Radiocarbon results

**FIGURE 4:**
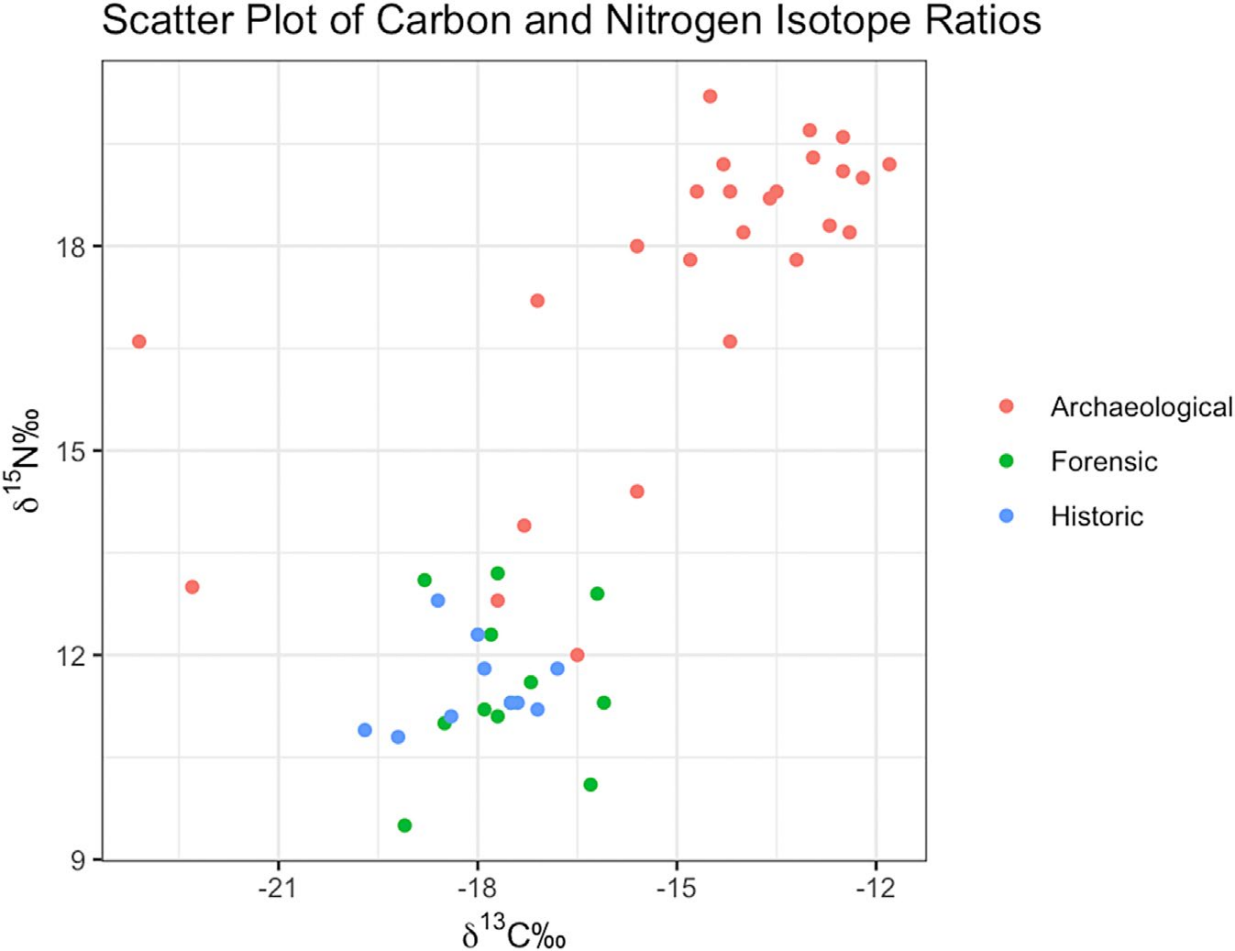
Scatterplot of carbon and nitrogen isotopes of human bone collagen, classified by conclusion—archaeological, historical, or forensic.

The dataset had radiocarbon dates ranging from modern to over 6000 BP. Full results with errors can be found in Data S2. In line with our hypothesis, we found that archaeological populations in most cases had marine‐based diets consistent with those reported in Schwarcz et al. [23]. However, there were also several samples that were considered historical which had distinct isotope values. Figure 4 is the scatterplot of the samples colored by which group they were assigned to. Importantly, historical and forensic samples had similar results, suggesting that dietary patterns shifted during the colonization of British Columbia, and has maintained similar since. We have termed this cluster of forensic and historical as “contemporary,” as they all share similar results and cannot be distinguished using carbon and nitrogen isotopes. Furthermore, there are four archaeological samples (study IDs 5, 9, 19, and 46) that had isotope values similar to the “contemporary” cluster; we assume that these individuals lived in the interior of British Columbia. However, as our binomial variable of interior or coastal is determined by the location of the reporting police department that does equate to location of origin.

The radiocarbon results support our original hypothesis, where dietary isotopes can be used to screen which cases are not of forensic interest. The cluster of cases similar to Schwarcz et al. (2014) have such a high degree of marine protein (likely marine mammal) that it is unlikely that any individual today in British Columbia would have results like this. Therefore, cases similar to this would not be sent for further testing, and instead, these remains are returned to community for reburial. While those that appear to have a terrestrial diet (5, 9, 19, 46) or deviate from the expected (i.e. 1 and 25) should continue to be investigated. In this circumstance, the next step is radiocarbon dating which is considerably more expensive and time intensive (Tables 2, 3, 4, 5).

**TABLE 2:** Descriptive results of radiocarbon dating.

Conclusion	*n*	Mean BP	$\surd \sum_{\left(\text{error}\right)}$	Interior	Coastal
Archaeological	26	1289	21.59	6	20
Historic	10	136	13.5	4	6
Forensic	12	F^14^C 1.2070	F^14^C 0.179	8	4

### 3.2 Logistic regression

The purpose of the logistic regression is to demonstrate a more objective probability‐based estimation of whether a sample is of a “contemporary” or archaeological context. As there are several cases that were archaeological but appeared “contemporary,” this model attempts to provide a probability‐based estimation of their class. As forensic and historical individuals share a similar diet, this model will not be able to effectively estimate beyond archaeological or not. Logistic regression is developed to estimate the probability between two outcomes; we have classified an archaeological context as = 1, and a contemporary context = 0.

The development of the logistic model began by regressing each independent variable against the categorical dependent variable. On their own, each variable did have statistically significant coefficients; however, when all three were combined into a singular model, there was no longer statistical significance. Models were compared using the Akaike information criterion (AIC) and Bayesian information criterion (BIC); for both measures, a smaller value indicates a better fit of the data (see Table 3).

**TABLE 3:** AIC and BIC comparison of five developmental models.

Model #	Variables in model	AIC	BIC	Coefficient's p
1	Interior vs. Coastal	60.446	64.0146	<0.01
2	*𝛿* ^13^C	49.387	52.9550	<0.001
**3**	***𝛿*** ^**15**^ **N**	**19.168**	**22.7360**	**<0.01**
4	*𝛿* ^15^N + *𝛿* ^13^C	21.005	26.3578	*𝛿* ^15^N < 0.01
5	*𝛿* ^15^N + *𝛿* ^13^C + Interior vs Coastal	19.629	26.7659	>0.05

*Note*: In bold is the selected model.

**TABLE 4:** Confusion matrix of training logistic model.

Training sample (*n* = 44)		Reference	
Predicted		“Contemporary”	Archaeological
	“Contemporary”	20	3
	Archaeological	0	21

**TABLE 5:** Confusion matrix of test samples in logistic model.

Test sample (*n* = 5)		Reference	
Predicted		“Contemporary”	Archaeological
	“Contemporary”	2	0
	Archaeological	0	2

**FIGURE 5:**
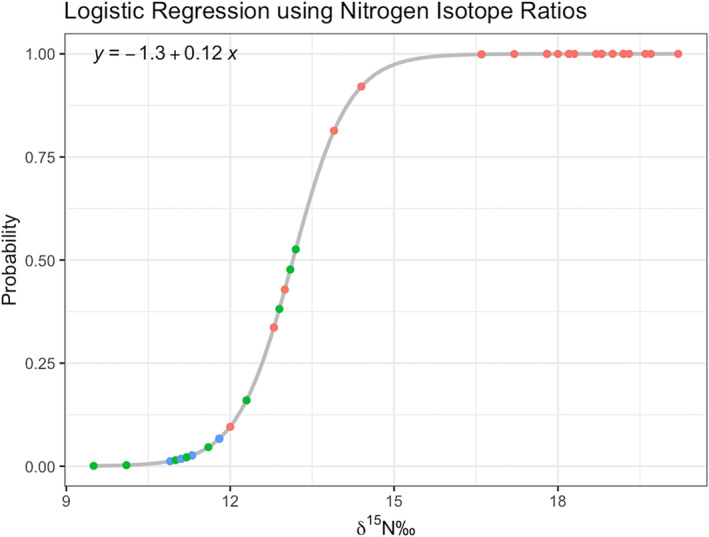
Plot of logistic regression curve. Nitrogen isotope values are used to provide a probability basis for whether a case will be estimated to be archaeological or contemporary in nature.

Of the five models developed, model 3 had the lowest AIC and BIC values. We suggest that this is due to the fractionation of nitrogen isotopes between trophic levels. As there is a 2‰–4‰ increase in *𝛿* ^15^N values between consumer and diet, this results in a large separation between a marine protein and terrestrial protein diet. As the two primary populations are largely separated by these different subsistence patterns, the classification using *𝛿* ^15^N values is parsimonious. As seen in Figure 4, most archaeological samples have significantly higher *𝛿* ^15^N than the historical and forensic cases. Importantly, there are several archaeological samples that appear to have a terrestrial diet; this overlap is beneficial for the predictive model. Logistic models have decreased power when there is a perfect separation in the independent variables. Therefore, having these archaeological cases that appear forensic increases the functionality of a predictive model. Figure 5 is a plot demonstrating the logistic regression curve and the fit of the data for the training regression. The model separates the data into archaeological (=1) and “contemporary” (=0); however, there are several cases that fall ~0.5 on the probability curve. Importantly, several of these cases are of forensic interest. Therefore, when using this logistic equation to evaluate its accuracy, and predict the class of each sample, we have moved the threshold for the archaeological classification to be above 0.75. We have chosen this because it decreases the probability of a false negative, where a forensic case is considered archaeological, and thus no longer investigated.

To evaluate the accuracy of this model, a test sample of five was removed from the original data. Using these test samples, confusion matrices were created to estimate the accuracy. Importantly, as there is a relatively large separation in the data for archaeological and contemporary cases, it is probable that there will be a very high accuracy. For datasets where there is increased overlap in the data for classes, the accuracy will likely be lower. As mentioned previously, it is important that the researcher determine what threshold is appropriate for their project. For this project, the risk associated with a false negative (a forensic case being estimated as archaeological) is significantly more problematic than a false positive. Therefore, we have increased the threshold (archaeological = >0.75%) to decrease the probability of this problem. In doing so, the accuracy for correctly estimating forensic cases increases, but the accuracy for archaeological cases (particularly those with terrestrial diets) decreases. As the goal with this method is to be a preliminary step in the death investigation of unidentified remains, additional archaeological samples being radiocarbon dated is preferable to forensic cases not being further investigated.

The accuracy of the training model was estimated to be 93% (95% CI: 81%–99%) and was statistically significant (*p* < 0.0001). However, as the same data are used to train this model, a follow‐up test sample was used to better predict it accuracy when new data are introduced.

In this test sample, it correctly identified the entire test sample. However, as noted previously, there is a large degree of separation between archaeological and “contemporary” cases. As this was a random subsample, we did not introduce cases where there may be incorrect prediction (terrestrial archaeological samples). The confidence interval for the test samples was 40%–100%. The large CI is likely due to the high degree of separation in the populations of the test sample. The correct classification of samples within the test sample was not statistically significant; this is because there would be comparable results/accuracy by flipping a coin. For future cases where there may be more heterogeneity, the significance would likely increase.

## 4 DISCUSSION

In this paper, we have demonstrated how isotopic analysis of carbon and nitrogen values in human bone collagen can be an effective predictor of whether a sample is archaeological or recent in age, and therefore medicolegally significant. This method is especially effective in British Columbia where the dietary differences between archaeological and contemporary populations is quite pronounced (terrestrial protein versus marine protein as a primary food type). This method will likely see decreased accuracy in regions where the dietary patterns between populations are more similar (e.g., we were unable to determine historical from forensic individuals in this study, as the dietary patterns were too similar). Isotopic methods can be applied as an early step in the death investigation of unidentified skeletonized human remains in regions where there are differences in diet between modern populations and archaeological. Similarly, Bartelink et al. [29] were able to use carbon isotopes and a linear discriminate function analysis to estimate whether a case was an American, or Asian soldier. As each population had a different reliance on C_4_ and C_3_ plant foods, their model had a high degree of accuracy. Thus, methods of discrimination using dietary isotopes can be effective if each population relies to some degree on different foodstuffs. Future analysis would benefit from using new isotope systems.

### 4.1 Scatterplot

With only the visual analysis of a scatterplot, it is possible to determine that there were at least two discrete populations, and with one population being very similar to the published *𝛿* ^13^C and *𝛿* ^15^N values for archaeological populations in British Columbia, it is clear that the remaining cluster should be further investigated. Importantly, the consideration of where these cases are coming from can be informative; for example, archaeological diets in the interior of British Columbia are much more similar to contemporary diets. Thus, samples coming from the interior may be archaeological when they appear contemporary. However, to avoid scenarios where cases of medicolegal significance are misidentified as archaeological, all cases that are similar to contemporary diets should be further investigated. As demonstrated here, radiocarbon dating is an effective next step to determine whether the samples are from a modern population, or historic. As radiocarbon dating is expensive, and often involves lengthy wait times, initial analysis of dietary isotopes of bone collagen can be an effective strategy to save resources and time.

### 4.2 Logistic regression

A logistic regression model was developed to provide a more objective means to estimate whether a sample is of forensic interest. There was a high degree of model accuracy; however, there is a question of whether it was necessary in this study area. In this circumstance, there is a large degree of separation between the populations in the entire dataset; thus, a predictive model may not be necessary. However, for circumstances where the populations in the data have a more similar dietary pattern, predictive models similar to this may be more productive.

### 4.3 Limitations

A limitation associated with this project is the small number of individuals with an archaeological terrestrial diet. With a larger sample of archaeological cases that had a terrestrial diet, a more complex model including more variables may have permitted a more accurate model. As seen in Figure 5, there were several forensic cases that had a 50/50 chance of belonging to either population. This is due to the similar *𝛿* ^15^N values between “contemporary” cases, and the few archaeological cases with a terrestrial diet. Therefore, to increase the confidence of these cases, more archaeological terrestrial samples and likely other isotope systems would make the model more accurate. Future analysis of different isotopes systems (such as lead or sulfur) that may vary between contemporary populations and historic populations may also provide a more fruitful analysis.

## 5 CONCLUSIONS

This paper has demonstrated how carbon and nitrogen isotope analysis of human bone collagen can be useful to coroner/medical examiner and police services in the early stages of death investigation in regions where there is clear isotopic separation in diets between modern and archaeological populations. In British Columbia, there is a large difference between the diets of contemporary populations (terrestrial diets) and archaeological populations (typically marine diets). Therefore, isotope analysis is an effective and resource‐efficient method to screen which cases are not of forensic interest early on in death investigation.

The analysis done in this study demonstrates how carbon and nitrogen isotope analysis of human bone collagen has the capacity to close death investigations prior to DNA analysis, radiocarbon dating, or other analytical methods. This paper presents over 10 years of collaboration, and how isotope analysis can regularly contribute to the determination of medicolegal significance.

## FUNDING INFORMATION

This research is funded by SSHRC and NSERC grants to M. Richards, and a CGS‐M SSHRC Grant for D. Tarrant.

## CONFLICT OF INTEREST STATEMENT

The authors have no conflicts of interest to declare.

## Supporting information


Data S1.

Data S2.

## Data Availability

All data are in the Appendix.
