# Supplementary material for: The use of dietary isotopes as a preliminary step in the death investigation of unidentified skeletal human remains in British Columbia, Canada
Source: J Forensic Sci. 2024 Nov 7;70(1):28–37. doi: 10.1111/1556-4029.15653 (PMC11693519; doi:10.1111/1556-4029.15653)
Supplement: Supplementary file 1 — Data S1. [file JFO-70-28-s001.docx]

SUPPLEMENTARY INFORMATION S1 Equipment, measurement standards and analytical uncertainty for the three isotope labs used in this study.

**(1) Simon Fraser University**

At Simon Fraser University, carbon and nitrogen isotopic and elemental compositions were determined using a Thermo Delta V Plus mass spectrometer coupled to a Thermo Flash Isolink elemental analyzer (Thermo-Finnigan, Thermo Scientific, Bremen, Germany) in the Department of Archaeology Isotope Laboratory (Simon Fraser University, Burnaby, Canada). Stable carbon and nitrogen isotope compositions were calibrated relative to VPDB (*δ*^13^C) and AIR (*δ*^15^N) using USGS40 and USGS41a(1, 2) (Table S1; 1).

TABLE S1.1 Standard international reference materials used for calibration of *δ*^13^C relative to VPDB and *δ*^15^N relative to AIR.

| Standard | Material | Accepted *δ*^13^C | Accepted *δ*^15^N |
| --- | --- | --- | --- |
|  |  | (‰, VPDB) | (‰, AIR) |
| USGS40 | Glutamic Acid | −26.39 | −4.52 |
| USGS41a | Glutamic Acid | +36.55 | +47.55 |

The following internal lab standards are included in each sample run to calculate precision and to monitor analytical uncertainty. The isotopic compositions reported here for internal standards represent long term (multi-year, > 1000 measurements) averages calibrated to VPDB and AIR with USGS40 and USGS41a.

TABLE S1.2 Standard internal reference materials used for to monitor internal accuracy and precision.

| Standard | Material | Average *δ* ^13^C  (‰, VPDB) | Average *δ* ^15^N  (‰, AIR) |
| --- | --- | --- | --- |

SRM-1 Bone Collagen -13.63±0.19 +17.45±0.13

SRM-2 Bone Collagen -24.19±0.11 +0.90±0.08

SRM-3 Bovine Collagen -15.75±0.24 +9.13±0.09

(commercial)

Casein Casein -27.25±0.17 +5.90±0.13

(commercial)

Fish gelatin Gelatin (commercial, -22.92±0.10 +4.26±0.12

certified value)

**(2) University of British Columbia** (lab no longer exists)

The UBC carbon and nitrogen isotopic and elemental compositions of extracted collagen were determined using an Elementar vario MICRO cube elemental analyzer coupled to an Isoprime isotope ratio mass spectrometer (Elementar Analysensysteme GmbH, Langenselboldin, Germany) in the former Department of Anthropology Archaeology Isotope Laboratory (University of British Columbia, Vancouver, Canada). Stable carbon and nitrogen isotope compositions were calibrated relative to VPDB (*δ*^13^C) and AIR (*δ*^15^N) using USGS40 and USGS41.

TABLE S1.3 Standard reference materials used for calibration of *δ* ^13^C relative to VPDB and *δ* ^15^N relative to AIR.

| Standard | Material | Accepted *δ* ^13^C  (‰, VPDB) | Accepted *δ* ^15^N  (‰, AIR) |
| --- | --- | --- | --- |
| USGS40 | Glutamic Acid | −26.39 | −4.52 |
| USGS41 | Glutamic Acid | +37.63 | +47.57 |

The following internal lab standards were used to monitor accuracy and precision and were included in each sample run. The isotopic compositions for these standards represent long-term averages based on the following number of analyses: *n*=195 (NIST 1577c), *n*=270 (SUBC−1), *n*=341 (MET) (as reported in Jaouen et al.(3)).

TABLE S1.4 Standard reference materials used for to monitor internal accuracy and precision.

| Standard | Material | Average *δ* ^13^C  (‰, VPDB) | Average *δ* ^15^N  (‰, AIR) |
| --- | --- | --- | --- |
| NIST 1577c | Bovine Liver | −17.51±0.10 | +8.15±0.15 |
| SUBC−1 | Bone Collagen | −13.67±0.11 | +17.39±0.14 |
| MET | Methionine | −28.60±0.08 | −5.04±0.15 |

**(3) Max Planck Institute for Evolutionary Anthropology** (lab no longer exists)

Isotope and elemental compositions of carbon and nitrogen were measured on a Thermo-Finnigan Flash 2112 elemental analyser coupled to a Delta XP IRMS (Thermo-Finnigan, Thermo Scientific, Bremen, Germany). Samples were measured relative to the IAEA N1, N2, CH6 and CH7 standards.

TABLE S1.5 Standard international reference materials used for calibration of *δ*^13^C relative to VPDB and *δ*^15^N relative to AIR.

| Standard | Material | Accepted *δ*^13^C | Accepted *δ*^15^N |
| --- | --- | --- | --- |
|  |  | (‰, VPDB) | (‰, AIR) |

IAEA N1 Ammonium Sulfate n/a +0.43

IAEA N2 Ammonium Sulfate n/a +20.41

IAEA CH6 Sucrose -10.45 n/a

IAEA CH7 Polyethylene Foil -32.14 n/a

The following internal standards were used to calculate precision and to monitor analytical uncertainty and were included in each run. The isotopic compositions reported here for internal standards represent long term averages (multi-year, >1000 measurements) calibrated to VPDB and AIR with CO2 and N2 reference gases calibrated to IAEA N1, N2, CH6, and CH7. Long term variations on both standards for *δ*^13^C and *δ*^15^N were both less than 0.2 ‰.

TABLE S1.6 Standard internal reference materials used for to monitor internal accuracy and precision.

| Standard | Material | Average *δ*^13^C | Averaged *δ*^15^N |
| --- | --- | --- | --- |
|  |  | (‰, VPDB) | (‰, AIR) |

Brad-001 Methionine -29.77 +2.22

NBS 1577b Bovine Liver -21.60 +7.65

**References**

1. Qi H, Coplen TB, Geilmann H, Brand WA, Böhlke JK. Two new organic reference materials for δ^13^C and δ^15^N measurements and a new value for the δ^13^C of NBS 22 oil. Rapid Communications in Mass Spectrometry. 2003;17(22):2483–7.

2. Qi H, Coplen TB, Mroczkowski SJ, Brand WA, Brandes L, Geilmann H, et al. A new organic reference material, l‐glutamic acid, USGS41a, for δ13C and δ15N measurements − A replacement for USGS41. Rapid Communications in Mass Spectrometry. 2016;30(7):859–66.

3. Jaouen K, Szpak P, Richards MP. Zinc isotope ratios as indicators of diet and trophic level in arctic marine mammals. PLoS One. 2016;11(3):e0152299.
